# Persistent intracranial hyper-inflammation in ruptured cerebral aneurysm after COVID-19: case report and review of the literature

**DOI:** 10.1186/s12883-023-03493-z

**Published:** 2024-01-02

**Authors:** Pin Fee Chong, Kanako Higashi, Wakato Matsuoka, Koichi Arimura, Yuhei Sangatsuda, Katsuma Iwaki, Yuri Sonoda, Yuko Ichimiya, Akiko Kamori, Akiko Kawakami, Soichi Mizuguchi, Noriyuki Kaku, Yasunari Sakai, Shouichi Ohga

**Affiliations:** 1 Department of Pediatrics, Graduate School of Medical Sciences, Kyushu University, 3-1-1 Maidashi, Higashi-Ku, Fukuoka, 812-8582 Japan; 2 Emergency and Critical Care Center, Kyushu University Hospital, Fukuoka, 812-8582 Japan; 3 Department of Neurosurgery, Graduate School of Medical Sciences, Kyushu University, Fukuoka, 812-8582 Japan; 4 Research Center for Environment and Developmental Medical Sciences, Kyushu University, Fukuoka, 812-8582 Japan

**Keywords:** Subarachnoid hemorrhage, SARS-CoV-2, Posterior cerebral artery, IL-6, Cerebral aneurysm

## Abstract

**Background:**

The systemic manifestations of coronavirus disease 2019 (COVID-19) include hyperinflammatory reactions in various organs. Recent studies showed evidence for the frequent involvement of central nervous system in affected patients; however, little is known about clinical features of cerebrovascular diseases in childhood-onset COVID-19.

**Case presentation:**

A 10-year-old boy recovered from SARS-CoV-2 infection without complication. On 14 days after infection, he presented with loss of consciousness. A head computed tomography detected a ruptured cerebral aneurysm at the left posterior cerebral artery accompanying subarachnoid hemorrhage (SAH). Immediate surgical intervention did not rescue the patient, resulting in the demise 7 days after admission. Serological and genetic tests excluded the diagnosis of vasculitis and connective tissue disorders. Retrospective analysis showed markedly higher levels of interleukin (IL)-1β, IL-6 and IL-8 in the cerebrospinal fluid than the serum sample concurrently obtained. A review of literature indicated that adult patients with COVID-19 have a risk for the later development of SAH during the convalescent phase of COVID-19.

**Conclusions:**

SAH is a severe complication of COVID-19 in children and adults who have asymptomatic cerebrovascular aneurysms. The markedly high levels of cytokines detected in the cerebrospinal fluid suggested that intracranial hyperinflammatory condition might be one of the possible mechanisms involved in the rupture of a preexisting cerebrovascular aneurysms.

## Introduction

The pandemic of COVID-19 continues to grow worldwide. To date, at least 760 million people have contracted severe acute respiratory syndrome coronavirus 2 (SARS-CoV-2) infection, and 6.8 million deaths have occurred since the first reported case in 2019 [1]. Although initially documented as a respiratory tract-targeted viral infection, SARS-CoV-2 has been associated with inflammatory reactions in systemic organs [2].

Accumulating evidence highlights the neurotropism of SARS-CoV-2, provided that an increasing number of reports show the association with cerebrovascular disease and neuroinflammatory conditions [3]. Hypercoagulability and cytokine release syndrome are also postulated as probable mechanisms of an increased susceptibility to cerebrovasular events in patients with COVID-19 [4].

Cerebrovascular events occur as intracerebral hemorrhage [5] and ischemic stroke [6]. Still rarer complications include cerebral venous sinus thrombosis and aneurysmal subarachnoid hemorrhage (SAH) [7, 8]. SAH might concurrently develop during the acute phase of COVID-19 in adult patients [9], but not reportedly in pediatric patients. We herein present a pediatric patient with ruptured intracerebral aneurysm after COVID-19.

## Case description

**Fig. 1:**
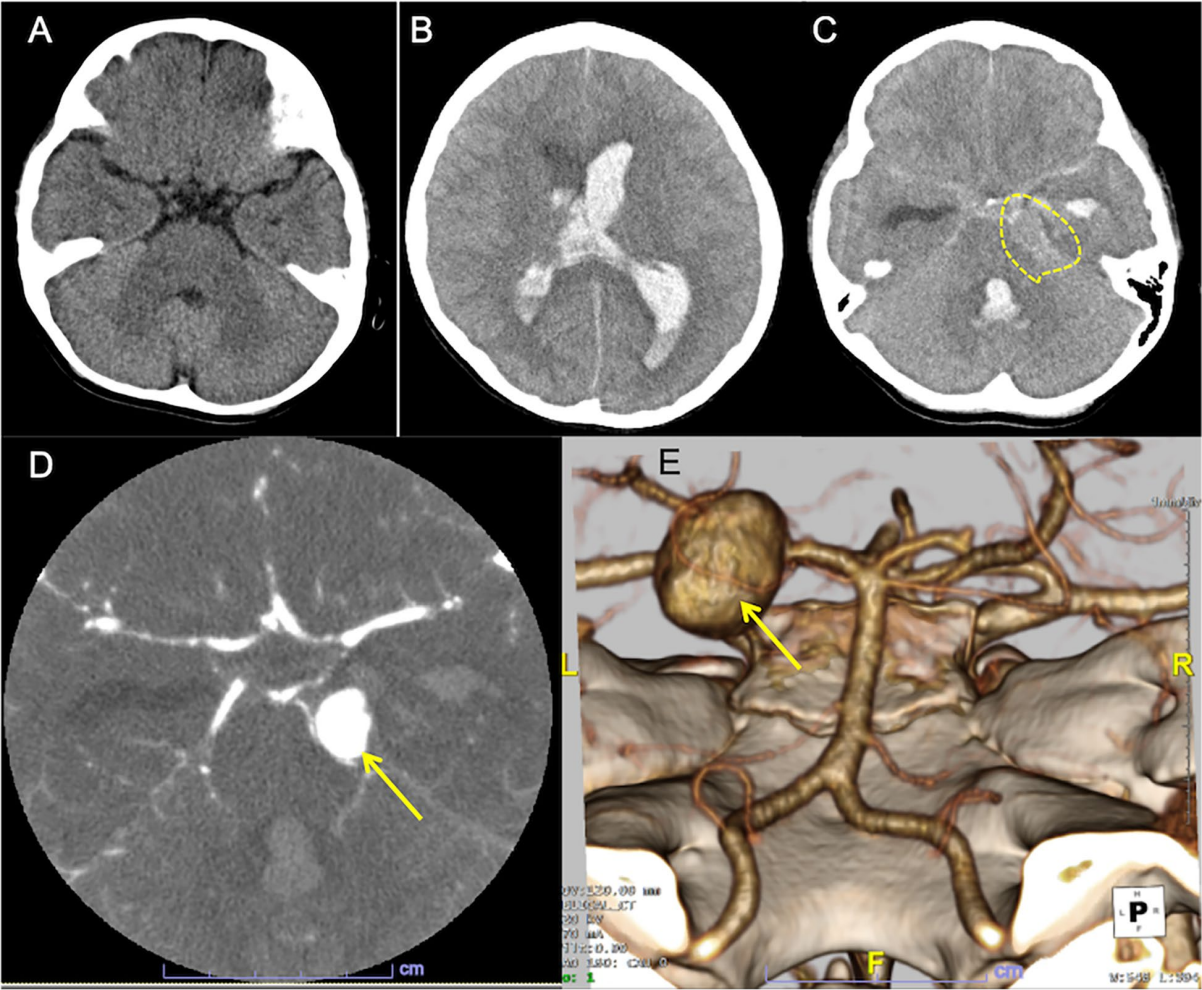
Computed tomography (CT) images. **A** A CT image taken at age one. No identifiable lesion suggestive of aneurysm. **B** Axial CT image on admission. The plain CT shows brain swelling, hydrocephalus with intraventricular hemorrhage. **C** Hyperdense material in the basal cistern, intraventricular hemorrhage in the left lateral and fourth ventricles, as well as a round lesion (encircled) at the left pontocerebellar cistern, indicating intracerebral aneurysm. **D** CT angiography reveals a large dome-shaped aneurysm (arrow) measuring 15 mm of the left posterior cerebral artery. **E** 3D-reconstruction image shows a saccular aneurysm in the left P2 segment (arrow)

A 10-year-old Japanese boy entered the emergency room, with complaints of headache and a loss of consciousness in the 2nd week of August, 2022. During this period, the Omicron BA.5 variant of SARS-CoV-2 was the most prevalent strain in Japan. He had no history of hypertension but self-limited infantile epilepsy at age one with normal neuroimaging and electroencephalography results. A computer tomography (CT) image at age one suggested no apparent aneurysmal lesion (Fig. 1A). Fourteen days before admission, he had fever and vomiting with positive test for SARS-CoV-2. After intravenous hydration, he obtained a prompt recovery on the next day. The child had not been previously vaccinated against COVID-19.

On admission, left-sided tonic seizure persisted and was terminated after infusions of diazepam. The Glasgow Coma Scale was E2V3M3. A polymerase chain reaction test on the nasopharyngeal sample was negative for SARS-CoV-2. An urgent head CT revealed hydrocephalus with massive intraventricular hemorrhage and the parenchymal edema (Fig. 1B). A hyperdense round-shape lesion was identified at the left pontocerebellar cistern, adjacent to the hyperdense material in the basal cistern (Fig. 1C). These indicated the diagnosis of aneurysmal SAH (WFNS clinical scale: Grade 4). An additional computed tomography angiography (CTA) detected a large saccular aneurysm sized 15 mm of the left posterior cerebral artery (PCA) (Fig. 1D). The 3D-reconstruction image confirmed a large aneurysm in the P2 segment (Fig. 1E).

**Fig. 2:**
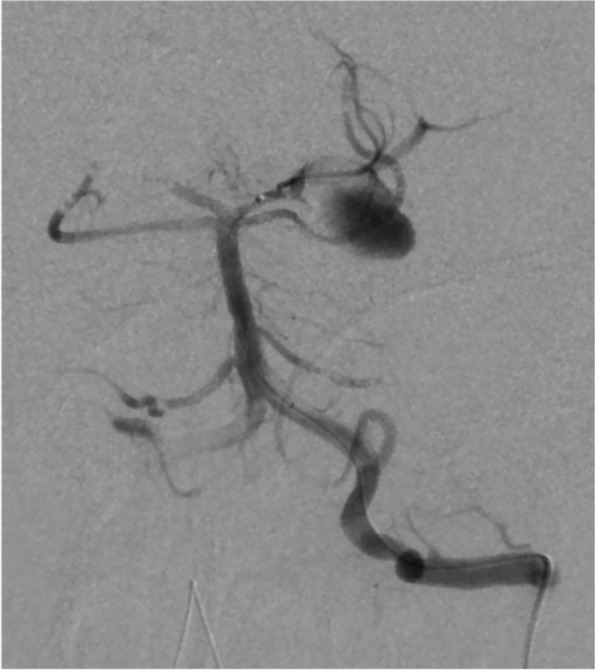
Cerebral digital subtraction angiography. Left vertebral angiography confirms the presence of left posterior cerebral artery aneurysm. Poor visualization of the arteries distal to basilar artery due to the increased intracranial pressure

Intensive care was started after immediate bilateral external ventricular drainage. Pupils began to dilatate from Day 2 of admission. The follow-up CT showed progressive brain edema. Left vertebral-angiography revealed poor perfusion to the arteries distal to basilar artery due to the increased intracranial pressure (Fig. 2). Additional interventions with coil occlusion and decompressive craniectomy did not reduce the intracranial pressure to less than 80 mmHg. He died on Day 7th of admission (21 days after the onset of COVID-19). Autopsy had not been performed following caregivers’ refusal to consent.

To exclude vasculitis or vasculopathy, we retrospectively analyzed cerebrospinal fluid (CSF) using “FilmArray” meningitis and encephalitis panel (Biofire Diagnostics, Utah, USA). The test was negative for 14 pathogens, including enterovirus and varicella zoster virus [10]. A genetic diagnostic panel for connective tissue disorders including Ehlers-Danlos syndrome, Marfan syndrome, Loeys-Dietz syndrome reported no pathogenic variants [11]. We measured concurrent cytokine concentrations in serum and the CSF with a flow-cytometric bead assay (BD Biosciences, San Jose, NJ). The CSF sample was obtained one day after the onset of SAH (15 days post-COVID-19) from ventricular drain 24 h after the procedure without evidence of rebleeding. Interleukin (IL)-6 level was > 750 times higher in the CSF showing 273,680 pg/mL compared to concurrently obtained serum sample measuring 356 pg/mL. CSF levels of IL-1β (CSF: 352 pg/mL; serum: 0 pg/mL) and IL-8 (CSF: 310,360 pg/mL; serum: 2,638 pg/mL) were also predominantly elevated. Tumor necrosis factor-α was undetectable in CSF or serum samples.

We reviewed literature on aneurysmal SAH and COVID-19 up to September 2022 (PUBMED search for the keywords: SAH, COVID-19 and aneurysm). We identified a total of 22 cases from 10 articles [7–9, 12–18]. The clinical information is summarized in Table 1. All but one adolescent case [12] were adult patients. The present patient was the youngest of all reported cases. The severity of COVID-19 was varied, ranging from asymptomatic to respiratory distress with systemic involvement (“Severe”). Only one patient had a previously detected aneurysm [17]. Twenty of 23 cases (87%) were SARS-CoV-2-positive during the acute stage of SAH. The remaining 3 (13%) including ours suffered from the later-onset SAH (> 2 weeks after infection). Aneurysmal size ranged from 1.4–21 mm (mean: 8.7 mm). The only one patient (Ref9-10) with PCA aneurysm showed a large aneurysmal size (21 mm) [9], as observed in our case.

**Table 1:** Summary of patients with aneurysmal subarachnoid hemorrhage during or after COVID-19

**No**	Patient	Age (years)	Sex	**COVID-19**			**SAH**		
				Severity^a^	Comorbidities	Duration (day)^b^	Signs at onset	Outcome	Reference
1	Ref12-1	13	Female	Severe	None	0	LOC	Severe disability	12
2	Ref13-1	NA (adult)	NA	NA	NA	3	NA	NA	13
3	Ref13-2	NA (adult)	NA	NA	NA	NA	NA	NA	
4	Ref13-3	NA (adult)	NA	NA	NA	9	NA	NA	
5	Ref16-1	31	Male	Mild	None	0	Headache, LOC	NA	16
6	Ref15-1	36	Female	Moderate	NA	1	Headache, LOC	Moderate disability	15
7	Ref14-1	60	Female	Severe	NA	0	LOC	NA	14
8	Ref9-1 to 10	< 30: 1 30–40: 5 40–50: 2 > 50: 2	Male: 5 Female: 5	Asymptomatic: 2 Mild: 2 Moderate: 1 Severe: 3	Hypertension: 1	0	NA	Good recovery: 7 Moderate disability: 1 Severe disability or death: 2	9
9									
10									
11									
12									
13									
14									
15									
16									
17									
18	Ref7-1	52	Male	Mild	Hypertension	0	LOC	Good recovery	7
19	Ref7-2	61	Male	Asymptomatic	Hypertension	14	LOC	NA	
20	Ref8-1	61	Female	Moderate	Hypertension, overweight	1	Headache, LOC	Good recovery	8
21	Ref17-1	68	Female	Asymptomatic	Previous aneurysm	0	Headache, vomiting	Good recovery	17
22	Ref18-1	58	Female	Severe	Colon cancer	21	LOC	Good recovery	18
23	**Present**	10	Male	Mild	None	14	LOC, seizure	Death	This study

*SAH* subarachnoid hemorrhage, *NA* not available, *LOC* loss of consciousness

^a^Severity represents respiratory distress syndrome with systemic involvement (severe), pneumonia with or without respiratory support (moderate) and mild respiratory symptoms or fever alone (mild)

^b^Each value indicates the duration from the diagnosis of symptomatic or asymptomatic SARS-CoV-2 infection to the onset of SAH

## Discussion

We report a pediatric patient with SAH resulting from a ruptured aneurysm at PCA 14 days after the recovery of COVID-19. Irrespective of geographical location, the incidence of aneurysmal SAH increased with age, and the mean age at onset was 47.4 years [19]. PCA-involving SAH accounts for 1% of all intracranial aneurysms [20]. Two combined unusual conditions discriminate this case from previously reported cases, suggesting a different patho-mechanism involved. The exclusion of other etiologies of SAH implied the association with a recent COVID-19 infection in this patient.

Cumulative evidence suggest COVID-19 predisposes patients to cerebrovascular events. SARS-CoV-2 virion established infection by binding to the angiotensin-converting enzyme 2 (ACE2), an enzyme critical for regulation of blood pressure and anti-atherosclerotic effects [21]. This may result in increased blood pressure, accelerating cerebral aneurysm formation in a short period. Additionally, COVID-19 induced endothelial damage activates the coagulation cascade [22], which may subsequently contribute to the rapid enlargement of aneurysms and consequently to their ruptures. The hyperinflammatory state in COVID-19 potentially exaggerates the increased permeability of the blood–brain barrier [15], causing elevations in matrix metalloproteinase-9 and subsequent arterial instability.

Despite a number of probable patho-mechanisms thus far proposed, the current knowledge concerning the connection between COVID-19 and SAH is limited in the real-world setting [23, 24]. In fact, there are conflicting data showing the incidence of SAH in patients with COVID-19. For example, a single institute reported that the incidence of SAH and intracerebral hemorrhage was increased during the pandemic period in 2020, compared to that in the reference period one year before [23]. Another study using the multivariate analysis did not find such an increase in the incidence, but did a higher mortality in patients with COVID-19 and SAH [24]. In our patient, the IL-6 level in CSF was not only increased to > 10-fold higher than those in patients with SAH [25], but also to > 100 folds of those with neuro-COVID [26]. Of note, the IL-6 level in CSF was much higher (> 750 times) than serum levels, suggesting that persistent intracranial inflammation raised a risk for the rupture of a preexisting aneurysm after COVID-19.

Childhood-onset SAH in a post-COVID-19 condition might be more than a coincidental finding, given unusual features in classically defined cases of SAH. Although the substantial impact of COVID-19 on the cerebral vasculature remains unknown, neurotropism and intracranial inflammation together with systemic vasculitis might additively contribute to the cerebrovascular vulnerability. Given that this is the first report showing the markedly increased levels of proinflammatory cytokines in CSF from a patient with post-COVID-19 SAH, further epidemiological and clinical studies are needed to rationalize the pathogenic link between COVID-19 and SAH in childhood.

## Data Availability

All datasets for this study are included in the manuscript.
